# Maximum gait speed and lumbar spinal mobility can affect quality of life in elderly women with lumbar kyphosis

**DOI:** 10.1016/j.xnsj.2022.100100

**Published:** 2022-01-16

**Authors:** Tatsuya Endo, Osamu Shirado, Ryoji Tominaga, Keita Sato, Takuya Miura, Masumi Iwabuchi, Toshikazu Ito

**Affiliations:** a Departments of Orthopaedic and Spinal Surgery, and Rehabilitation, AMEC (Aizu Medical Center) at Fukushima Medical University, Aizuwakamatsu, Fukushima, Japan; b Hokkaido Chitose College of Rehabilitation, Chitose City, Hokkaido, Japan

**Keywords:** Kyphosis, Pain-specific quality of life, Physical performance, Gait speed, Lumbar spinal ROM, Oswestry disability Index

## Abstract

**Background:**

The site and angle of kyphosis are important factors that affect quality of life (QOL). Lumbar kyphosis, rather than thoracic kyphosis, is reported to affect the QOL in patients with kyphosis. Increased angle of kyphosis in elderly people is associated with a decline in motor and physical functions, and also correlates with reduced QOL. Investigation of how physical performance affects their QOL would be helpful in developing an effective physical therapy program for elderly patients with kyphosis. The aims of the current study were to evaluate the physical performance including back muscle strength, spinal range of motion, and walking ability in elderly patients with lumbar kyphosis, and to examine its association with back pain-specific QOL.

**Methods:**

The design of this study is a cross-sectional study in a single hospital. A total of 51 elderly women aged 65 years or older diagnosed with kyphosis were enrolled in the study. The items evaluated were back pain (visual analog scale [VAS]), back-pain specific QOL (the Oswestry Disability Index [ODI]), maximum gait speed, thoracic kyphosis angle, lumbar lordosis angle, sacral inclination, spinal inclination, trunk extension/flexion range of motion (ROM), thoracic spinal ROM, lumbar spinal ROM, and back muscle strength. Data were analyzed using bivariate analyses and multivariate regression analyses.

**Results:**

Significant positive correlations were observed between ODI and VAS (rs=0.506) or spinal inclination (rs=0.626). Significant negative correlations were observed between ODI and maximum gait speed (rs=-0.664), lumbar lordosis angle, (rs=-0.553), trunk extension ROM (rs=-0.571), lumbar spinal ROM (rs=-0.651), or back muscle strength (rs=-0.521). Multiple regression analysis demonstrated that maximum gait speed (standard partial regression coefficient; b=0.484) and lumbar spinal ROM (b=0.463) had a significant impact on ODI. The results of analysis of variance were significant with R^2^ of 0.622.

**Conclusions:**

The current study demonstrated that maximum gait speed and lumbar spinal ROM influenced back-pain specific QOL in the elderly women with lumbar kyphosis. Maximum gait speed and lumbar spinal ROM should be evaluated thoroughly to effectively perform non-operative treatment in elderly people with lumbar kyphosis.

**Tweet**

Lumbar mobility and walking speed are predictive of changes in back-pain specific quality of life for patients with lumbar kyphosis.

## Introduction

Among adult spinal deformities, age-related spinal kyphosis is estimated to affect 20–40% of elderly people and is becoming one of the major medico-social problems in aging populations. [1,2] The site of kyphotic deformity is clinically significant. A previous study [3] pointed out that lumbar kyphosis, rather than thoracic kyphosis, affected the QOL in the patients with kyphosis. The overall incidence of lumbar degenerative scoliosis in patients between the ages of 50 and 80 is high but reported clinically relevant spinal deformity in only about 10% [4]. The most common physical finding is a loss of lumbar lordosis, progressing first to flat-back and in a smaller number of cases to frank lumbar kyphosis.

Most studies regarding the factors associated with back-pain specific QOL in patients with kyphosis have focused only on the angle of kyphosis. A study in patients over the age of 18 years has pointed out that worsening kyphosis angle is associated with health-related QOL (SF-36, SRS-22) [5]. It has also been reported that subjects with an increased kyphosis angle in the lumbar spine have a significantly lower QOL (SRS-29 and the Oswestry Disability Index [ODI]) than those with normal or lordotic lumbar angle [6]. Additionally, an increase in kyphosis angle is associated with a decrease in QOL regarding the satisfaction with subjective health, family relationships, economic conditions [7]. Kyphosis in the elderly deteriorates with many factors such as osteoporosis, osteoporotic vertebral compression fracture, disc degeneration, and other skeletal aging [8]. In addition to those factors, physical performance including trunk muscle strength and spinal mobility is closely correlated to kyphosis in the elderly [3,9,10]. However, no studies have investigated the relationship between physical performance and back-pain specific QOL in patients with kyphosis.

The purposes of the current study were to evaluate the relationship between back-pain specific QOL and physical performance that includes back muscle strength, spinal mobility, and walking ability in elderly patients with kyphosis, and to identify the factors affecting the QOL. Our hypothesis was that trunk extensor strength and/or extension mobility would affect the QOL in such patients.

## Subjects and methods

### Study design

This study was conducted in a cross-sectional design. Data and research protocol were approved by the research ethics committee of our institution. The patients were given the right to opt-out of the study.

### Patient recruitment

Patients who suffered from chronic low back pain for at least 3 months and were diagnosed with spinal kyphosis between September 2016 and June 2018 in our outpatient clinic were enrolled in this study. Spinal kyphosis was diagnosed by attending orthopaedic spine surgeons (R.T, M.I, and O.S), based on standing whole spinal radiographs using the Scoliosis Research Society-Schwab adult spinal deformity classification [11]. Spinal kyphosis was defined as a sagittal vertical axis (SVA: distance between C7 plumb line and posterosuperior corner of the S1 vertebral body) of 40 mm or more. All of the patients were women aged 65 years or older. Patients were excluded if they had fresh osteoporotic vertebral fractures, previous history of spinal surgery, radicular symptoms, inflammatory disease, malignancy, paralysis, primary joint disease such as active rheumatoid arthritis, moderate to severe musculoskeletal disease (i.e. knee, hip, and sacrum), severe psychiatric disturbance, or other serious complications.

### Data collection

First, the body height and weight of each patient were measured to calculate the body mass index (BMI). Back pain was evaluated using the visual analogue pain scale (VAS). The back-pain specific QOL was evaluated using the ODI [12], a widely used measure for evaluating the influence of back pain on QOL. In the present study, we excluded questions on sex life, thus a total of nine items were evaluated with a maximum possible score of 45 points. Higher scores indicate higher levels of disturbance.

Next, the following measurements were performed: gait speed, spinal alignment, range of motion (ROM) of the trunk, thoracic and lumbar spine, as well as back muscle strength.

### Assessments

#### Gait speed

Maximum gait speed was assessed using a 10-meter walk test [13]. Three meters were provided at the beginning and end of the 10-meter walkway to allow participants to accelerate and decelerate to help reduce gait variability. Maximum gait speed was measured twice consecutively by a therapist who accompanied each subject.

#### Measurement of spinal alignment

**Fig. 1:**
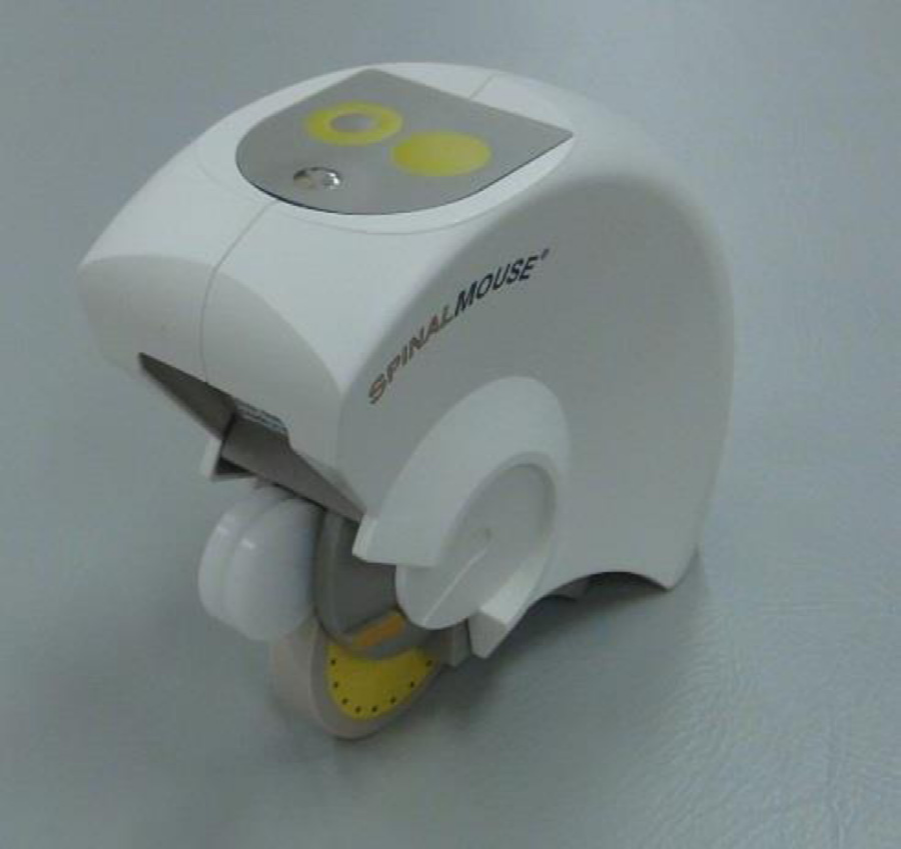
Spinal alignment measurement device (Spinal Mouse®).

**Fig. 2:**
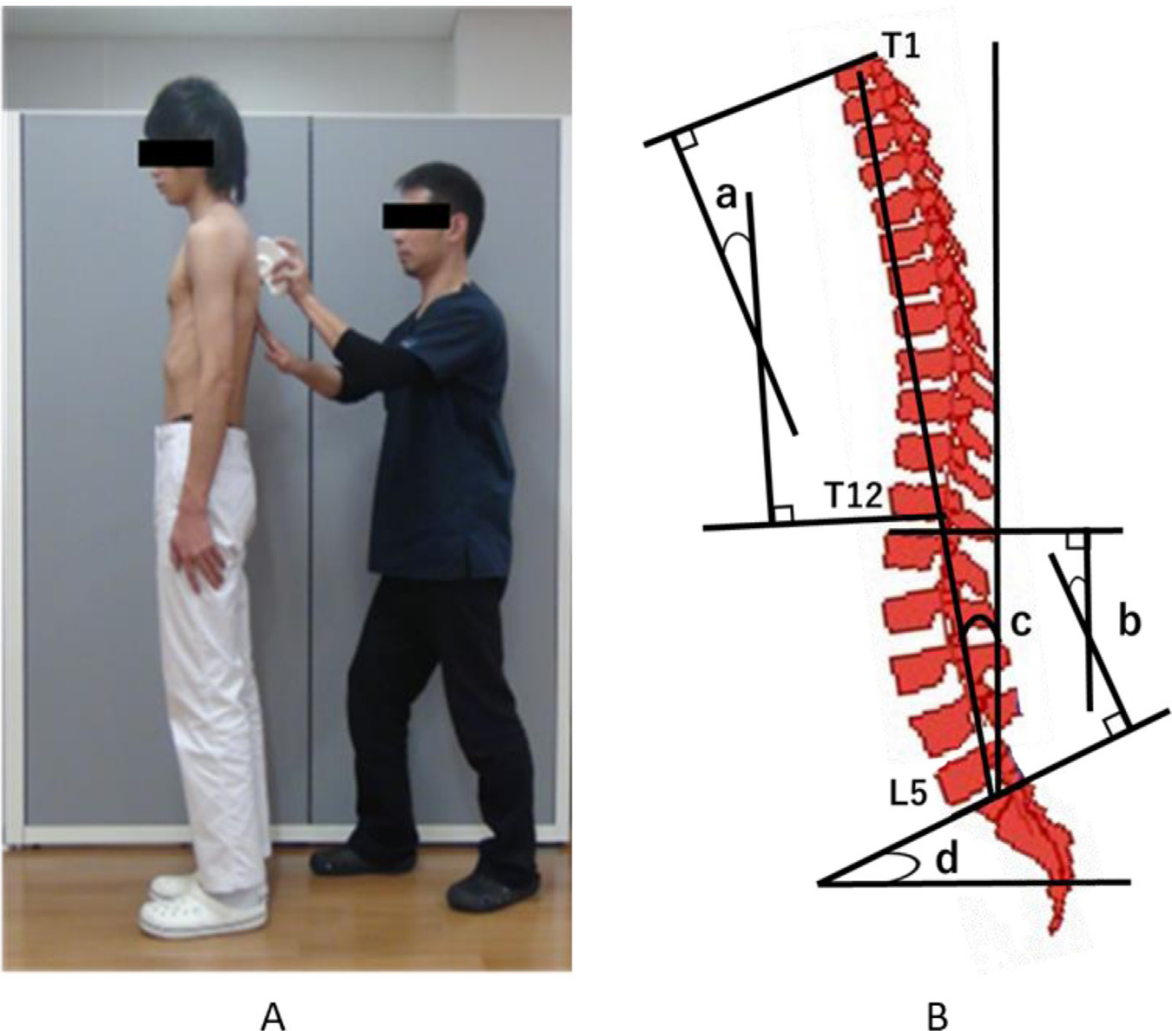
Measurement of spinal alignment on the sagittal plane. A. The scene of the measurement using Spinal Mouse® B. Each measured angle a. thoracic kyphosis angle, b. lumbar lordosis angle, c. spinal inclination angle, d. sacral inclination angle.

Spinal alignment in the sagittal plane was measured using the Spinal Mouse® (Index Ltd., Tokyo, Japan) (Figure 1). Its validity and intra-/inter-rater reliability have been verified to be as high as those obtained with radiography [14,15]. Measurements were performed with the subjects standing at rest in a natural and relaxed posture. The Spinal Mouse® was moved caudally from the seventh cervical vertebra to the third sacral vertebra along the spinous processes following the prescribed method (Figure 2A). The evaluation items included the thoracic kyphosis angle (TK; T1–T12), lumbar lordosis angle (LL; L1–L5), spinal inclination angle, sacral inclination angle (Figure 2B). All measurements were performed by carefully keeping the Spinal Mouse® in close contact with the spinous processes.

#### ROM measurements of the trunk, and thoracic and lumbar spine

**Fig. 3:**
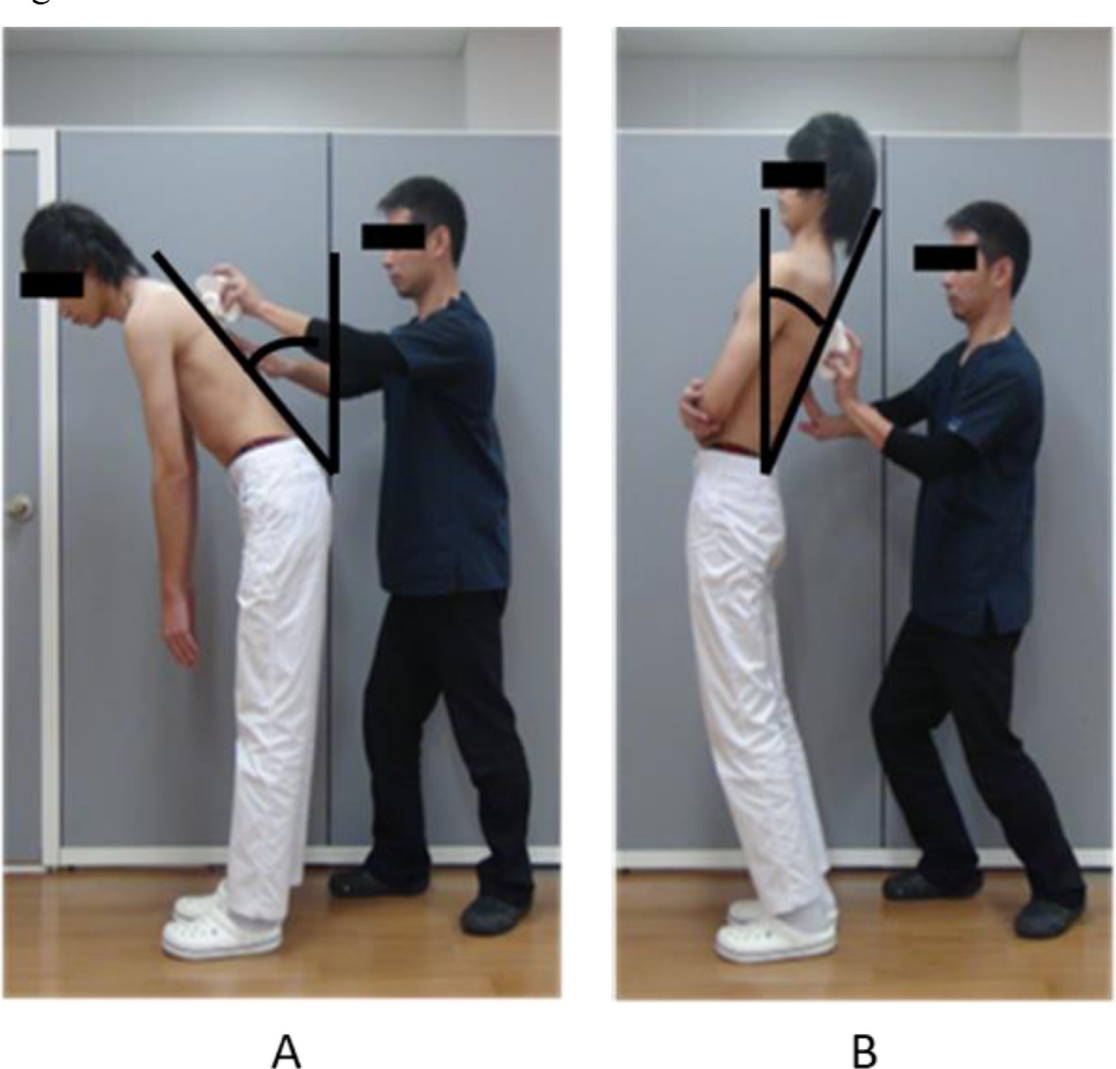
Measurements of the ROM for the trunk, and thoracic and lumbar spine. A. Maximum trunk flexion position B. Maximum trunk extension position.

The Spinal Mouse was used for the measurement. Trunk flexion ROM and extension ROM were obtained as the angle of spinal inclination during maximum trunk flexion and maximum trunk extension, respectively. Thoracic spinal ROM was obtained as the difference in the thoracic kyphosis angles measured in the maximum flexion position and in the maximum extension position. Lumbar spinal ROM was obtained as the difference in the lumbar lordosis angles measured in the maximum flexion position and in the maximum extension position. Each angle was measured three times (Figure 3). Maximum trunk flexion position was taken with the arms hanging straight down. Maximum trunk extension position was taken with the arms folded across the chest.

#### Measurement of back muscle strength

**Fig. 4:**
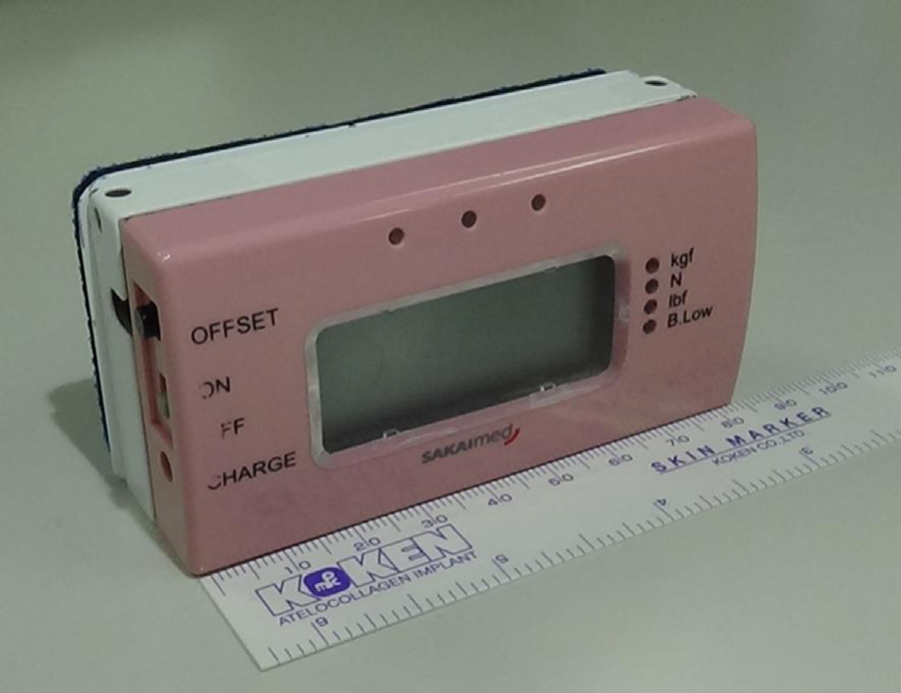
Back muscle strength measurement device (Mobie MT-100).

**Fig. 5:**
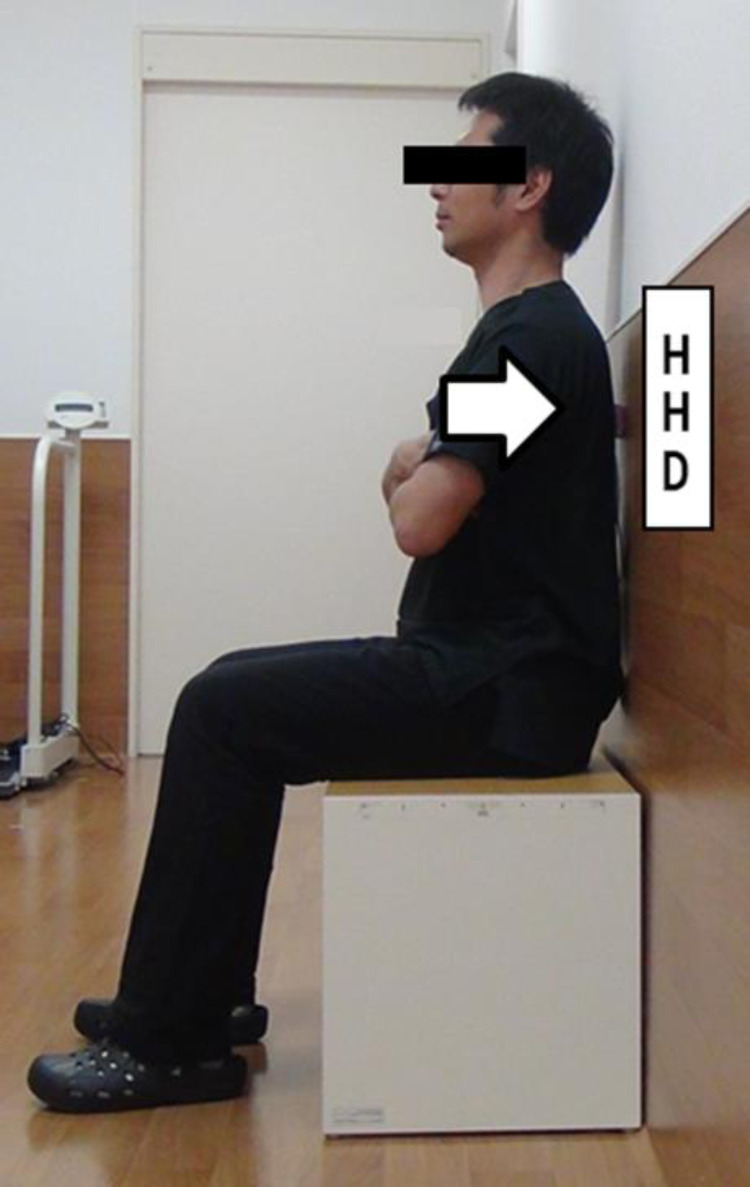
The scene of the measurement of back muscle strength.

Back muscle strength was measured using a hand-held dynamometer (HHD; Mobie MT-100; SAKAI Medical Co., Ltd. Tokyo, Japan; Figure 4) of which reliability and reproducibility have been verified in literature [16]. The measurements were performed on the subject while seated on a 40-cm high platform with the ankles and knees flexed to 90°. While the pelvis was not immobilized, the arms were folded across the chest. The soles of the feet were in contact with the floor. The patients were asked to apply pressure on the HHD device, which was placed between the wall and the patients’ back [17,18] (at the Th7 level, Figure 5). Under these conditions, two isometric contractions of 3 seconds each were performed. An average of two measurement results were used in the analysis. If the measured strength level increased by more than 10% from the first attempt, patients were asked to perform one additional attempt. The subjects received a full explanation of the method prior to measurements and practiced the motions. Fatigue was taken into consideration by allowing rest periods of at least 30 seconds between the measurements.

### Statistical analysis

Baseline characteristics are described using appropriate summary statistics. Spearman's rank-order correlation coefficient (rs) was calculated for correlations of ODI with age, BMI, back pain, maximum gait speed, thoracic kyphosis angle, lumbar lordosis angle, spinal inclination, sacral inclination, thoracic spinal ROM, lumbar spinal ROM, trunk ROM and back muscle strength. Stepwise multiple regression was used to investigate the factors affecting the back-pain specific QOL. When the ODI total score was the dependent variable, significantly correlated factors with increasing ODI were determined as independent variables. R Commander (Version 2.8.1, freeware) was used for statistical analysis. The significance level was 5%.

## Results

A total of 51 patients were included in this study. Table 1 shows the baseline data including age, BMI, back pain, ODI, maximum gait speed, thoracic kyphosis angle, lumbar lordosis angle, spinal inclination, sacral inclination, thoracic spinal ROM, lumbar spinal ROM, trunk ROM, and back muscle strength. The mean SVA was 110.4mm (SD: 33.8). The mean TK and LL was 28.6° (SD: 20.1) and -14.9° (SD: 19.8), respectively. This means that the subjects in this study have distinctive lumbar kyphosis.

**Table 1:** Baseline data of all patients BMI, body mass index; SVA, sagittal vertical axis; VAS, visual analog pain scale; ROM, range of motion; ODI, Oswestry disability index; SD, standard deviation. Data are expressed as mean and SD.

Variables	Mean	SD
Age (years)	75.0	8.0
BMI (kg/m^2^)	23.1	3.0
SVA (mm)	110.4	33.8
Back pain (VAS) (mm)	48.8	17.4
ODI score	36.9	15.4
Maximum gait speed (m/s)	0.99	0.31
Thoracic kyphosis angle (°)	28.6	20.1
Lumbar lordosis angle (°)	-14.9	19.8
Spinal inclination (°)	13.9	13.6
Sacral inclination angle (°)	-5.7	9.8
Trunk flexion ROM (°)	109.4	16.3
Trunk extension ROM (°)	3.1	20.5
Thoracic spinal ROM (°)	18.5	11.4
Lumbar spinal ROM (°)	45.0	19.1
Back muscle strength (kgf)	11.5	3.5

### Correlations of ODI with measured values

The mean ODI score was 36.9 (SD: 15.4) and ODI score (Section 1, Pain Intensity) was 2.5 (SD: 1.3). Significant positive correlations were observed between ODI and VAS (rs=0.506) or spinal inclination (rs=0.626). Significant negative correlations were observed between ODI and maximum gait speed (rs=-0.664), lumbar lordosis angle, (rs=-0.553), lumbar spinal ROM (rs=-0.651), trunk extension ROM (rs=-0.571), or back muscle strength (rs=-0.521) (Table 2).

**Table 2:** Correlations of ODI with clinical variables BMI, body mass index; VAS, visual analog pain scale; ROM, range of motion. Spearman's rank-order correlation coefficient (rs) was calculated for correlations of ODI. Significance was defined by a p-value < 0.05 and significant values are italicized.

Variables	Rs	p
Age (years)	0.193	0.298
BMI (kg/m^2^)	-0.049	0.792
Back pain (VAS) (mm)	0.506	*0.004*
Maximum gait speed (m/s)	-0.664	*<0.001*
Thoracic kyphosis angle (°)	-0.070	0.709
Lumbar lordosis angle (°)	-0.553	*0.001*
Spinal inclination (°)	0.626	*<0.001*
Sacral inclination angle (°)	-0.005	0.697
Trunk flexion ROM (°)	-0.058	0.755
Trunk extension ROM (°)	-0.571	*<0.001*
Thoracic spinal ROM (°)	-0.138	0.459
Lumbar spinal ROM (°)	-0.651	*<0.001*
Back muscle strength (kgf)	-0.521	*0.003*

### Factors affecting ODI

There was no multicollinearity among the independent variables (|rs| >0.9). Hence, the variables exhibiting significant correlations with ODI were used for analysis. The stepwise multiple regression analysis showed that maximum gait speed (standard partial regression coefficient; b=0.484) and lumbar spinal ROM (b=0.463) had a significant impact on ODI. The results of analysis of variance were significant with R^2^ of 0.622, showing a high degree of fit (Table 3).

**Table 3:** Associations of ODI with clinical variables ROM, range of motion; b, standardized partial regression coefficient (beta). Stepwise multiple regression was used to investigate the factors affecting back-pain specific QOL. Significance was defined by a p value < 0.05. The total model explained 62.2% of variance in ODI (R^2^=0.622).

Variables	Coefficient (b)	P
Maximum gait speed (m/s)	0.484	<0.001
Lumbar spinal ROM (°)	0.463	<0.001
R^2^	0.622	

## Discussion

The subjects in the current study were demonstrated to have distinctive lumbar kyphosis, which has been reported to affect QOL more than thoracic one [3,6]. The site of the kyphosis can be important in understanding the pathology.

The results of the current study demonstrated that, for the first time, maximum gait speed and lumbar spinal ROM were closely associated with back-pain specific QOL in the patients with lumbar kyphosis. So far, most studies investigating factors associated with back-pain specific QOL in patients with kyphosis have focused solely on the severity of kyphosis. The increased angle of kyphosis in elderly people has been shown to be related to reduced physical abilities such as walking [12], balance [12], [13], [14], and back muscle strength [13,14], thus resulting in worsened QOL [5], [6], [7]. However, there have been few studies evaluating the direct association between physical performance and back-pain specific QOL in the elderly with lumbar kyphosis.

### Management of kyphosis

Adult spinal deformity including kyphosis can be managed non-operatively or surgically. Despite little strong supporting evidence, non-surgical treatments are regarded as the first-line treatment therapy. Among such treatments, therapeutic exercise is one of the major treatment modalities with a goal of managing pain and maintaining function [8]. Surgery is one of the most effective treatments in the patients with adult spinal deformity (ASD) including spinal kyphosis. However, it can cause morbidity and mortality during and/or after the surgery. The surgery for ASD especially has high complication rates such as pseudarthrosis, instrumentation failure, and proximal junctional fracture. We do not believe that it should be indicated for all the patients.

The treatment of spinal deformity is not only radiological correction by surgery. QOL can be improved even without radiological correction by surgery. Surgery is the only option for those who want correction, while therapeutic exercise is recommended for those for whom cosmetic problem is not the main complaint. Among non-operative treatments, therapeutic exercise can be a promising treatment. It has been recently suggested as a preoperative treatment, or *prehabilitation* [[20], [21]], as well, which is beneficial for surgical candidates for spinal kyphosis. One of the goals in the current study was to obtain useful information for providing exercise treatment. A self-reported QOL assessment is helpful for understanding the patient's condition.

X-ray or CT images represent only one aspect of structural deformity. Combining those images with subjective measures such as ODI may provide clinicians with a more complete insight into the effects of spinal deformity on patients. Therapeutic exercise targets the management of pain and dysfunction to improve QOL. Surgery may be unnecessary if QOL improves with exercise therapy. To improve the effectiveness of exercise therapy, it is important to identify the physical functions that affect QOL.

### Association between gait speed and lumbar spinal ROM and back-pain specific QOL

Assessment of physical performance plays an important role in planning and performing practical therapeutic exercise regimen for elderly patients with kyphosis. In conjunction with our results, the evaluation of the maximum gait speed and lumbar spinal ROM seems to be essential when exercises are considered as a treatment modality. Gait speed is associated with activity of daily living, physical function [[22], [23]] and health-related QOL [24], and its measurement has been used to predict life function for geriatric patients [25]. Gait speed is a comprehensive performance parameter that reflects muscle strength and balance ability. Therefore, gait speed is important for elderly patients with lumbar kyphosis.

Spinal ROM can also be an important factor that affects daily life [3]. The current study showed that a decrease in lumbar spinal ROM was associated with a decline in QOL. In addition, trunk extension ROM was strongly correlated with back-pain specific QOL (rs=-0.571). ROM in the extension direction can be particularly important since the postural balance is likely to be lost when standing or walking with reduced extension ROM. The decreased ROM of the lumbar spine is assumed to worsen QOL by restricting the degree of freedom in various postures and activity of daily living. Therapeutic exercise is important in that it can preserve lumbar ROM. Long fusion surgery including whole lumbar spines with spinal instrumentation results in no motion in the lumbar spine.

### Strengths and limitations

The current study had several strengths. First, this is the first survey to investigate the association between back-pain specific QOL and various physical performances, rather than the degree of kyphosis. In this study, we investigated the relationship (not correlation) between physical function and QOL as independent factors adjusted for confounding factors. Second, gait speed was selected first as a factor that affects back-pain specific QOL in the multivariable analysis. Although it has been previously reported that walking ability is decreased in patients with kyphosis [19], the current study revealed a relationship between maximum gait speed and QOL in kyphosis. Third, spinal mobility was measured separately for the thoracic and lumbar spine. As a result, the ROM of the lumbar spine was found to have an impact on back-pain specific QOL. A previous study [3] pointed out that lumbar kyphosis, rather than thoracic kyphosis, affected the QOL in Japanese patients with kyphosis. The present study further showed that the spinal ROM, especially lumbar spinal ROM, was more important than spinal alignment. These findings suggest the focus be on the lumbar spinal ROM in the therapeutic exercise program.

Despite the strengths of our study, there were several limitations. First, selection bias was present. Although kyphotic deformity was targeted, lumbar kyphosis was the main disorder in the actual subjects. According to the ODI score, the subject's QOL was moderately impaired. Therefore, this study examined the clinical significance regarding lumbar kyphosis with moderately impaired QOL. Second, although we found a correlation between back-pain specific QOL and physical performance in the patients with lumbar kyphosis, we could not conclude a causal relationship, as this was a cross-sectional study. Third, the present study did not evaluate environmental or psychological factors which might have affected the QOL. The physical performance and QOL of patients with kyphosis are likely to be affected by a variety of factors, such as mental states and activity. Fourth, we measured trunk flexion and extension ROM only in the standing position. No assessments in other postures in motion were performed. The measurement under a moving condition such as walking should be investigated in future. A prospectively designed research is needed to further clarify the actual relationship between physical performance and the disease-specific/ general QOL in patients with kyphosis.

## Conclusion

In conclusions, we evaluated the physical performance that influenced the back-pain specific QOL in the elderly patients with kyphosis. The subject of the current study had distinctive lumbar kyphosis. Maximum gait speed and lumbar spinal ROM strongly affected the back-pain specific QOL. Maximum gait speed and lumbar spinal ROM should be evaluated thoroughly to better understand how physical performance affects QOL in elderly patients with lumbar kyphosis.

## Declaration of Competing Interest

Authors declare that they have no conflict of interest.
